# Sulindac Enhances the Killing of Cancer Cells Exposed to Oxidative Stress

**DOI:** 10.1371/journal.pone.0005804

**Published:** 2009-06-05

**Authors:** Maria Marchetti, Lionel Resnick, Edna Gamliel, Shailaja Kesaraju, Herbert Weissbach, David Binninger

**Affiliations:** 1 Department of Biological Sciences, Florida Atlantic University, Boca Raton, Florida, United States of America; 2 Center for Molecular Biology and Biotechnology, Florida Atlantic University, Boca Raton, Florida, United States of America; Bauer Research Foundation, United States of America

## Abstract

**Background:**

Sulindac is an FDA-approved non-steroidal anti-inflammatory drug (NSAID) that affects prostaglandin production by inhibiting cyclooxygenases (COX) 1 and 2. Sulindac has also been of interest for more than decade as a chemopreventive for adenomatous colorectal polyps and colon cancer.

**Principal Findings:**

Pretreatment of human colon and lung cancer cells with sulindac enhances killing by an oxidizing agent such as tert-butyl hydroperoxide (TBHP) or hydrogen peroxide. This effect does not involve cyclooxygenase (COX) inhibition. However, under the conditions used, there is a significant increase in reactive oxygen species (ROS) within the cancer cells and a loss of mitochondrial membrane potential, suggesting that cell death is due to apoptosis, which was confirmed by Tunel assay. In contrast, this enhanced killing was not observed with normal lung or colon cells.

**Significance:**

These results indicate that normal and cancer cells handle oxidative stress in different ways and sulindac can enhance this difference. The combination of sulindac and an oxidizing agent could have therapeutic value.

## Introduction

Sulindac was one of the early non-steroidal anti-inflammatory drugs (NSAIDs), which affect prostaglandin production by inhibiting cyclooxygenases (COX) 1 and 2 [1]. For more than a decade, sulindac has also been of interest as a chemopreventive treatment for adenomatous colorectal polyps and colon cancer [2]–[5], especially in patients with familial adenomatous polyposis [6]. Sulindac has also been reported as a chemopreventive agent for mouse urinary bladder cancer [7]. The anti-tumorigenic activity of sulindac against colon cancer may involve both COX inhibition [2] and activities that are independent of COX inhibition [8]–[11]. It has been reported that sulindac induces apoptosis of colon cancer cells, [11], [12], which appears to involve changes in gene expression [12]–[18].

Sulindac is a pro-drug that must be converted to the active COX-inhibitor, sulindac sulfide [19]. We have previously shown that conversion of sulindac to sulindac sulfide can be catalyzed by MsrA, a member of the methionine sulfoxide reductase (Msr) family of enzymes [20]. The Msr system has been studied in detail in recent years, after it was shown that MsrA may play a role in aging and age related diseases [21]–[23]. The obvious function of the Msr system is to reduce methionine sulfoxide (Met(o)) in proteins back to methionine (Met) (reviewed in [23]), although it also functions as part of a ROS scavenger system, in which the Msr system permits Met residues in protein to function as catalytic antioxidants [24]. Support for the scavenger role of MsrA has come from recent studies with both PC-12 neuronal cells, in which MsrA was overexpressed [25], and human lens cells, in which MsrA expression was down regulated [26]. Thus, there is considerable evidence to suggest that the Msr system plays an important role in protecting cells against oxidative damage.

Since sulindac is a substrate for MsrA [20], it seemed reasonable that the killing of cancer cells by sulindac might involve oxidative stress. Additionally, we wanted to determine whether normal cells and cancer cells responded in a similar way(s) after sulindac treatment and oxidative stress. In a preliminary study, we showed that treatment of a squamous cell cancer cell line with sulindac and an oxidizing agent led to nearly a 500% increase in intracellular ROS levels and significant cell death. In contrast, normal human epidermal keratinocytes did not show an increase in ROS levels or cell death. These results led to a limited clinical trial that showed promising potential of using topical application of sulindac and hydrogen peroxide for treatment of actinic keratoses [27].

In the present studies we extended these earlier results using cancer cell lines derived from lung and colon tissue. We provide further evidence that the enhanced killing observed with sulindac and oxidative stress involves mitochondrial dysfunction leading to cell death via apoptosis. These new data strengthen the potential for specifically enhancing the therapeutic application of sulindac and its derivatives for cancer treatment by using them in conjunction with a compound that produces reactive oxygen species (ROS).

## Results

### Sulindac enhances the killing of tumor cells by oxidative stress, but does not involve either COX inhibition or the Msr system

Human lung and colon cancer cell lines were preincubated in the presence or absence of sulindac for 48 hours. Excess sulindac was removed by washing prior to the 2 hr incubation with TBHP as described in the Materials and Methods. Sulindac was used at 500 µM final concentration since preliminary experiments using sulindac at this concentration showed no significant effect on cell viability for either of the cancer cell lines.

**Figure 1:**
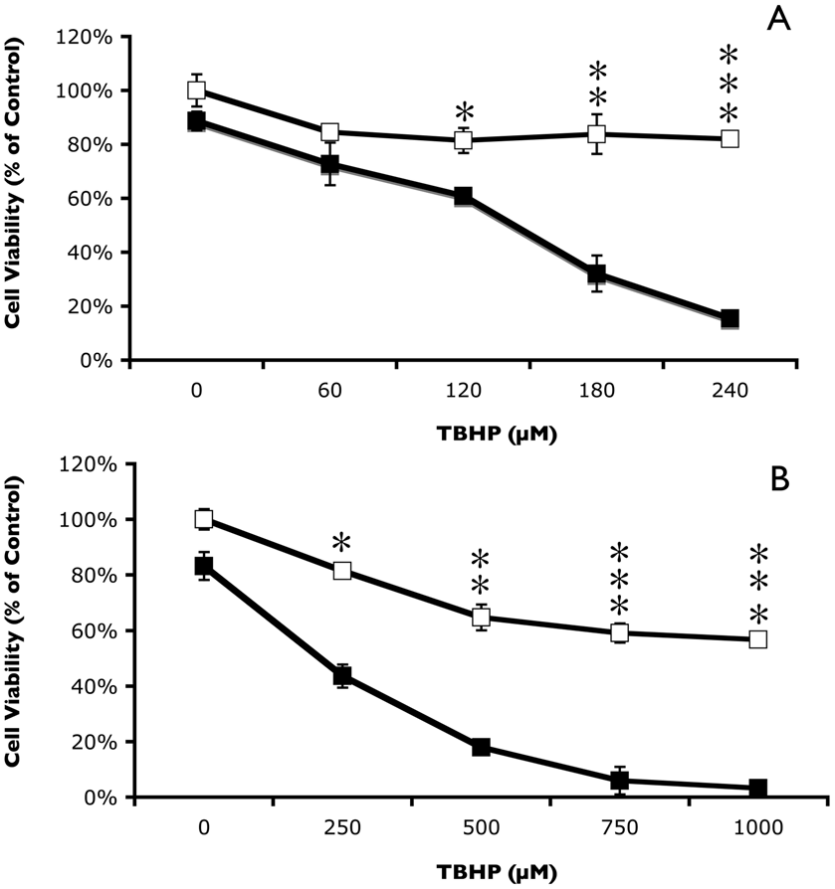
Effect of sulindac on the viability of cancer cells in response to oxidative stress. Lung cancer cells (A) or colon cancer cells (B) were incubated in the presence (▪) or absence (□) of 500 µM sulindac for 48 hr. Cells were then washed to remove the free sulindac prior to incubation for 2 hr with the indicated concentration of TBHP and cell viability was measured using the MTS assay described in Materials & Methods. Cell viability is expressed as % of control (cells not pretreated with sulindac or exposed to TBHP). Error bars are standard error of the mean (SEM) expressed as a % of the mean value of four replicate samples from a representative experiment. Significance of the differences between cells treated with and without sulindac, but exposed to the same concentration of TBHP: *p<0.01; ** p<0.001; *** p<0.0001.

**Figure 2:**
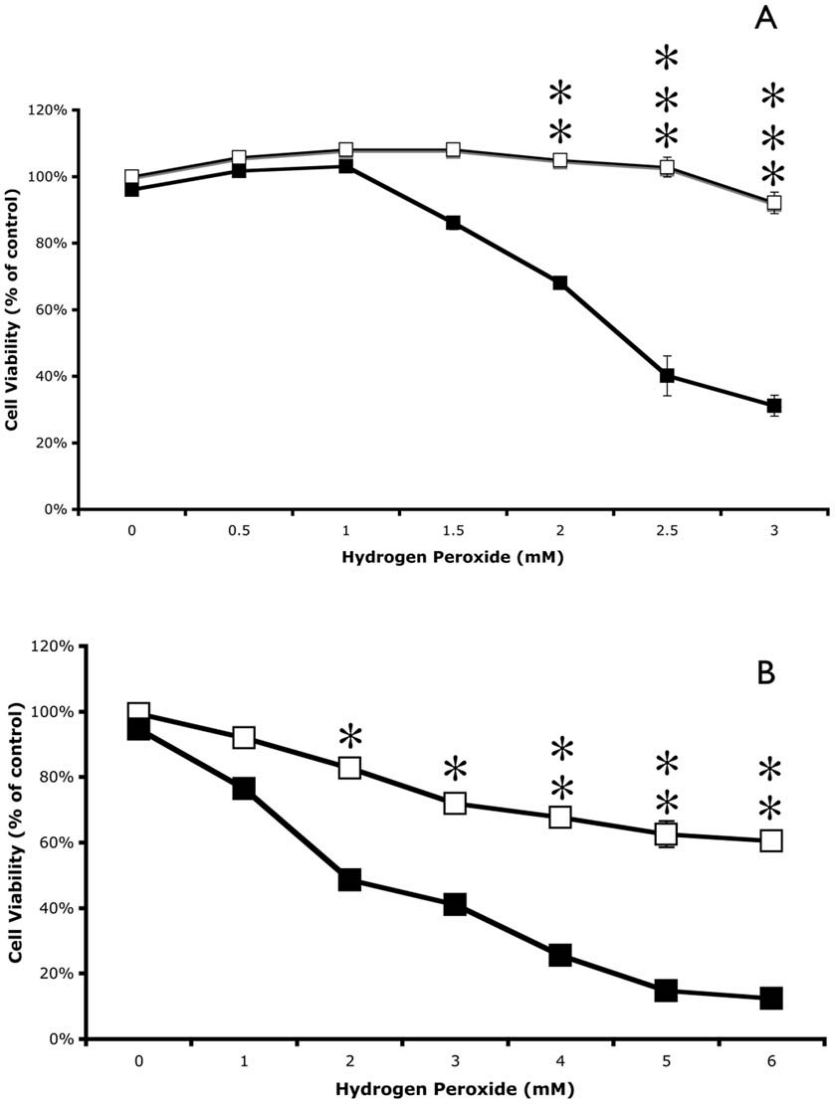
Effect of sulindac on the viability of cancer cells in response to hydrogen peroxide. Lung cancer cells (A) or colon cancer cells (B) were incubated in the presence (▪) or absence (□) of 500 µM sulindac for 48 hr. Cells were then washed to remove the free sulindac prior to incubation for 2 hr with the indicated concentration of hydrogen peroxide. Cell viability was measured using the MTS assay described in Materials & Methods. Cell viability is expressed as % of control (cells not pretreated with sulindac or exposed to hydrogen peroxide). Error bars are standard error of the mean (SEM) expressed as a % of the mean value of four replicate samples from a representative experiment. Significance of the differences between cells treated with and without sulindac, but exposed to the same concentration of hydrogen peroxide: *p<0.01; ** p<0.001; *** p<0.0001.

Each cancer cell line had a marked decrease in cell viability in the presence of TBHP following pretreatment with 500 µM sulindac (Figure 1A and 1B). Viability of lung cancer cells pretreated with sulindac was reduced by greater than 80% following incubation for 2 hr with 240 µM TBHP when compared to control cells that were not pretreated with sulindac (Figure 1A). Similar responses to TBHP were observed with sulindac treated colon cancer cells (Figure 1B), although a higher concentration of TBHP was required for significant killing of the colon cancer cells. Sulindac also enhanced the killing of both cancer cell lines when TBHP was replaced with hydrogen peroxide, at concentrations between 1.0 mM and 6.0 mM (Figure 2).

**Figure 3:**
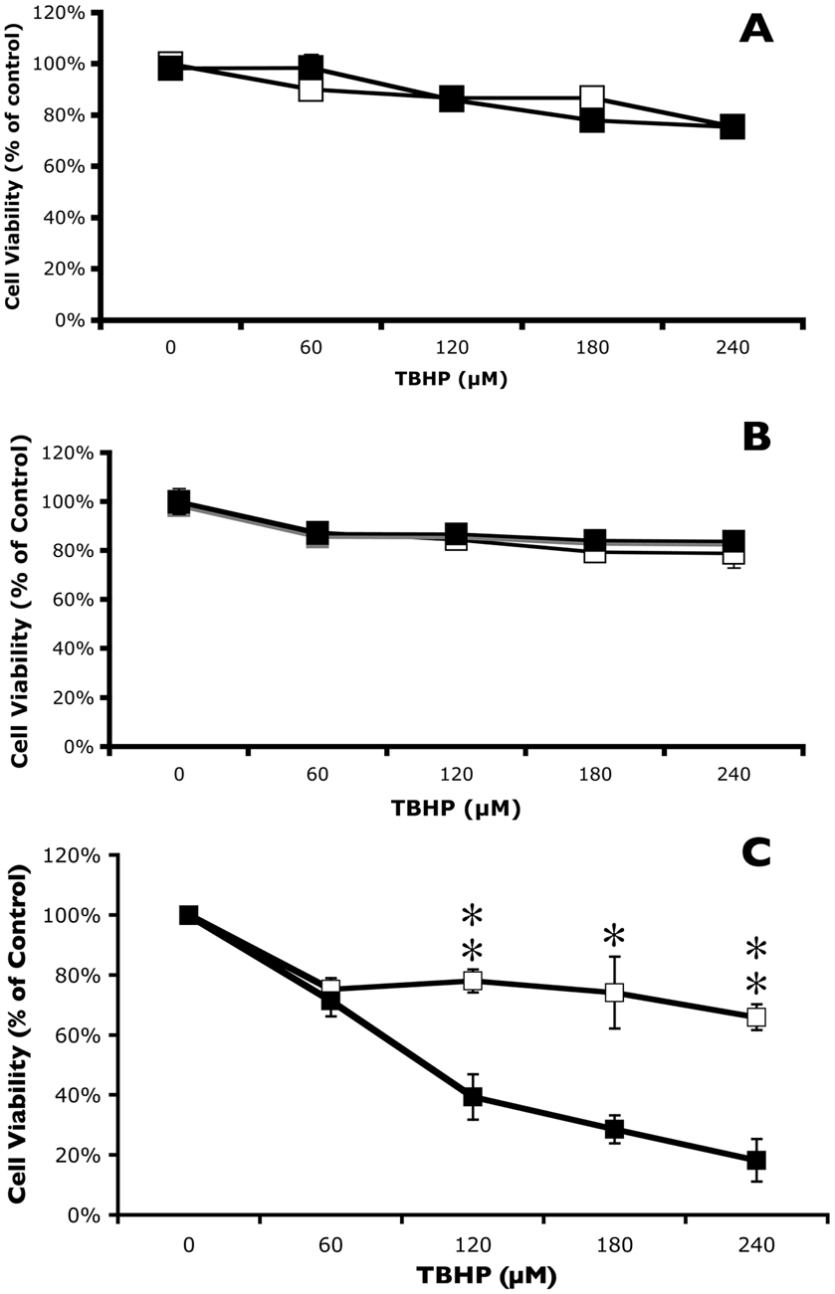
Effect of other NSAIDs or sulindac sulfone on the viability of lung cancer cells in response to oxidative stress. Lung cancer cells were incubated in the presence (▪) or absence (□) of either 500 µM acetylsalicylic acid (A), 500 µM ibuprofen (B) or 250 µM sulindac sulfone (C) for 48 hr. Cells were then washed to remove the free NSAID or sulindac sulfone prior to incubation for 2 hr with the indicated concentration of TBHP. Cell viability was measured using the MTS assay described in Materials & Methods. Cell viability is expressed as % of control (cells not exposed to an NSAID, sulindac sulfone, or TBHP). Error bars are standard error of the mean (SEM) expressed as a % of the mean value of four replicate samples from a representative experiment. Significance of the differences between cells treated with and without sulindac sulfone, but exposed to the same concentration of TBHP: *p<0.01; ** p<0.001; *** p<0.0001.

Several lines of evidence indicate that the enhanced killing of cancer cells by sulindac and oxidative stress does not involve COX inhibition. Two other NSAIDs, acetylsalicylic acid (aspirin) and ibuprofen, at 500 µM, failed to increase the sensitivity of cancer cells to oxidative stress (Figure 3A and 3B, respectively). As noted above, sulindac is a pro-drug that must be converted to the active COX-inhibitor, sulindac sulfide [19], primarily through the activity of MsrA [20]. To determine whether the enhanced killing of sulindac-treated cancer cells by TBHP involved reduction of sulindac to sulindac sulfide, the active inhibitor of cyclooxygenases, the experiments described above were repeated using sulindac sulfone, which is not a substrate for MsrA (unpublished data) or a COX inhibitor [28]. Because of increased toxicity of sulindac sulfone a lower concentration (250 µM) was used for these experiments. Pretreatment of lung cancer cells with 250 µM sulindac sulfone (Figure 3C), followed by exposure to TBHP gave comparable results to those seen using 500 µM sulindac (compare Figures 1A and 3C). Similar results using sulindac sulfone were also obtained with the colon cancer cells lines (data not shown). Thus, the collective data indicate that the increased sensitivity of sulindac treated cancer cells to oxidative stress, under the conditions used, does not involve either the Msr system or COX inhibition.

### Sulindac does not enhance the killing of normal cells exposed to oxidative stress

**Figure 4:**
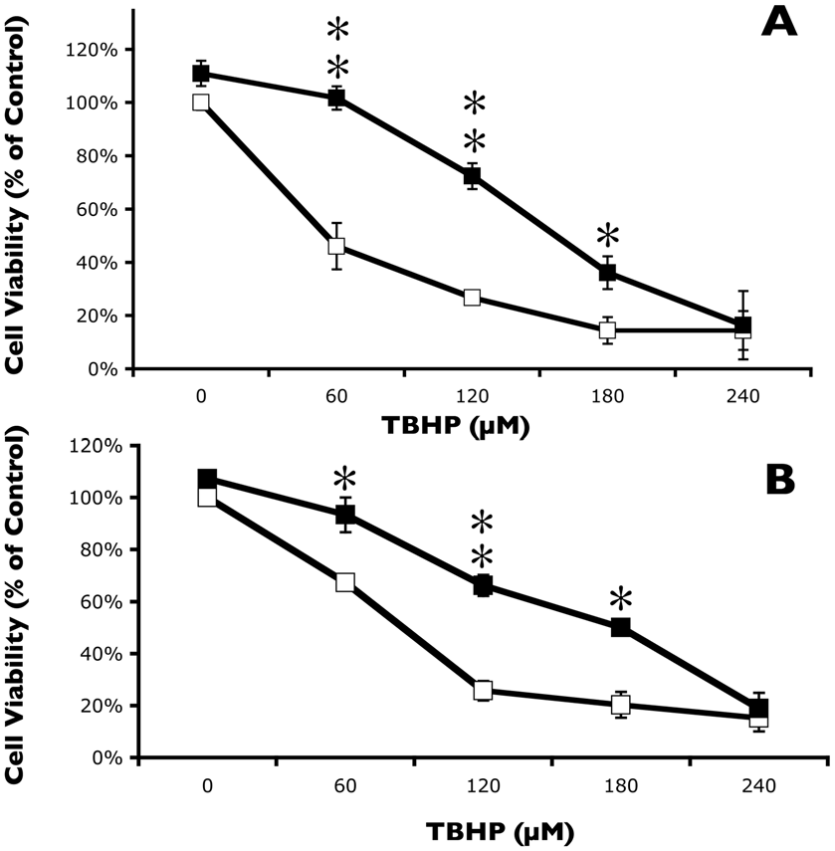
Sulindac and sulindac sulfone protect normal lung cells against oxidative stress. Normal lung cells were incubated for 48 hr in (A) the presence (▪) or absence (□) of 500 µM sulindac or (B) the presence (▪) or absence (□) of 250 µM sulindac. See Materials and Methods and legend to Figure 1 for further details. Significance of the differences between cells treated with and without sulindac or sulindac sulfone, but exposed to the same concentration of TBHP: *p<0.01; ** p<0.001; *** p<0.0001.

It was important to determine whether the enhanced killing effect of sulindac and sulindac sulfone on cancer cells in the presence of TBHP (Figure 1) also occurred with normal, non-immortalized cells. Figure 4 shows the effect of pretreating normal lung cells with sulindac or sulindac sulfone on cell viability after oxidative stress using TBHP. Incubation of normal lung cells with 500 µM sulindac for 48 hr prior to exposure to TBHP not only did not enhance killing, but sulindac provided protection from oxidative stress caused by TBHP (Figure 4A). The protective effect from oxidative stress on normal lung cells was also observed when cells were pretreated with sulindac sulfone (Figure 4B).

Similar experiments were performed using the normal colon cells. There was no effect on cell viability in the presence of TBHP when the normal colon cells were pretreated with either 500 µM sulindac or 250 µM sulindac sulfone (data not shown). Thus, neither normal lung nor normal colon cells showed enhanced killing by TBHP following treatment with sulindac or sulindac sulfone, as was observed with the two cancer cell lines.

### Sulindac pretreatment of lung cancer cells leads to elevated levels of ROS and loss of mitochondrial membrane potential

**Figure 5:**
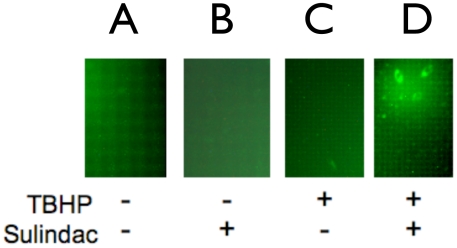
Intracellular ROS levels in lung cancer cells pretreated with sulindac followed by oxidative stress. The panels show intracellular ROS fluorescence. (A) untreated cells; (B) cells treated with only sulindac; (C) cells treated with only TBHP; (D) cells treated with both sulindac and TBHP. The incubation conditions are described in Figure 1. The cells were prepared for fluorescence microscopy and the green fluorescence signal was quantified as described in Materials and Methods. Fluorescence levels are the average of two independent experiments and were adjusted for the percentage of viable cells. The SEM was less than 10% for each sample. The increase in fluorescence compared to control cells in panel A are: B, 53%; C, 57%; D, 401%.

Lung cancer cells were used to gain more information on the mechanism of the enhanced killing effect of sulindac in the presence of TBHP. To investigate whether the enhanced killing of the cancer cells observed with sulindac and oxidative stress might involve mitochondrial dysfunction, changes in the level of intracellular ROS were determined. For these experiments lung cancer cells were treated with sulindac for 48 hours, exposed to TBHP and then the intracellular ROS level was visualized using a fluorescent dye as described in the Materials and Methods. The results are shown in Figure 5. Compared to untreated lung cancer cells (Figure 5A), cells treated with sulindac alone (Figure 5B) or TBHP alone (Figure 5C) showed a modest increase (53–58%) in ROS levels based on appearance of green fluorescence. However, lung cancer cells that were pretreated with 500 µM sulindac followed by a 2 hr incubation with 80 µM TBHP (Figure 5D) had a 400% increase in intracellular green fluorescence compared to untreated cells (compare Figure 5A and 5D). These data clearly show that pretreatment of the lung cancer cells with sulindac leads to a large increase in intracellular ROS following exposure to oxidative stress, supporting the results on skin cancer cells reported recently [27].

**Figure 6:**
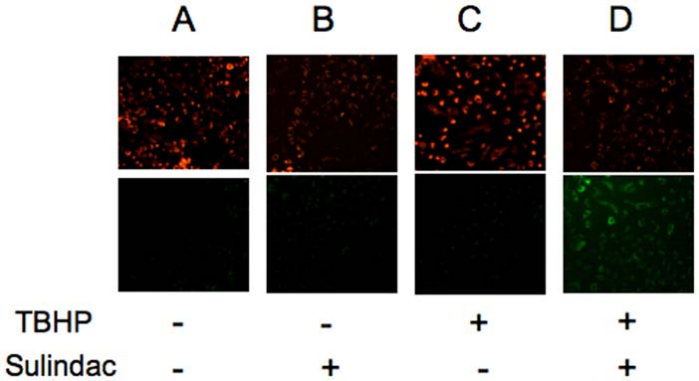
Mitochondrial membrane potential as measured by JC-1 distribution in lung cancer cells. Upper panels show red fluorescence images while lower panels show green fluorescence images. Loss of mitochondrial membrane potential was detected by a decrease of red fluorescence with a concomitant increase of green florescence. The experimental design is described in the legends of Figure 1. (A) untreated cells; (B) cells treated with only sulindac; (C) cells treated with only TBHP; (D) cells treated with both sulindac and TBHP. The cells were prepared for fluorescence microscopy and the fluorescence signal was quantified as described in Materials and Methods. Results are an average of three independent experiments. SEM was less than 10% for each sample. Quantitative analysis of green fluorescence is expressed as follows in arbitrary units: panel A –1.39; panel B –1.23; panel C –1.29; panel D –25.3.

To further explore the mechanism of killing cancer cells exposed to oxidative stress after pretreatment with sulindac, we investigated whether there is a concomitant loss of mitochondrial membrane potential, which is known to initiate apoptotic cell death. Effects on mitochondrial membrane potential were evaluated using changes in the fluorescence of the JC-1 dye as described in Materials and Methods. The results are shown in Figure 6. The top panels show red fluorescent images and the lower panels green fluorescent images. Loss of mitochondrial membrane potential would result in decreased red fluorescence and a corresponding increase in green fluorescence. Relative to untreated cells (Figure 6A) or cells treated with only sulindac (Figure 6B) or TBHP alone (Figure 6C), sulindac pretreatment of lung cancer cells followed by oxidative stress (Figure 6D) resulted in disruption of mitochondrial membrane potential as evidenced by nearly a 20-fold increase in green fluorescence (see legend of Figure 6 for additional details). In summary, pretreatment of the lung cancer cells with sulindac followed by treatment with TBHP leads to a marked increase in intracellular ROS and a significant loss of mitochondrial membrane potential. These results indicated that cell death was occurring via apoptosis, which was confirmed by Tunel analysis (Supplementary Figure S1)

## Discussion

Sulindac and its metabolites, such as sulindac sulfide and sulindac sulfone, have been shown to have anti-cancer activity [29]. Consistent with our previous study of skin cancer cells [27], we have shown that the killing of lung and colon tumor cell lines can be enhanced significantly if sulindac is combined with an oxidant, such as TBHP or hydrogen peroxide. We also have shown that sulindac pretreatment can enhance the killing of skin cancer cells caused by arsenic trioxide (data not shown), which kills cancer cells by generating intracellular ROS, as reported elsewhere for lung cancer cells [30]. It seems reasonable that sulindac may enhance the efficacy of any anticancer drug where the mechanism of action involves oxidative damage. The successful application of a multiple drug therapy has been recently reported for a clinical trial involving almost 300 patients at risk for recurrence of colorectal adenomas who were treated with a combination of sulindac and difluoromethylornithine, an inhibitor of polyamine synthesis [31].

It appears likely that the mechanism of the selective killing of cancer cells seen in these studies involves mitochondrial dysfunction, possibly as a result of increased ROS production. While treatment of cancer cells with sulindac or TBHP individually leads to a modest increase in the level of ROS (Figure 5B and [27], [32]), there is a dramatic increase in the intracellular levels of ROS in cells pretreated with sulindac and then exposed to TBHP (Figure 5D). In addition, there is a significant disruption of mitochondrial membrane potential under the same experimental conditions (Figure 6), suggesting that sulindac causes apoptosis in cancer cells exposed to oxidative stress. The anticancer effect of sulindac alone has been reported to involve apoptotic death [33]. The results with sulindac sulfone and other NSAIDs indicate that the effect of sulindac in our system does not involve COX inhibition or the Msr system.

The present results indicate a fundamental difference in the way normal and cancer cells respond to oxidative stress. Sulindac and its metabolites can accentuate this difference, which leads to enhanced killing of cancer cells, but not normal cells, by oxidative stress. It is well established that cancer and normal cells differ in their oxidative metabolism and that cancer cells have a higher rate of glycolysis than normal cells, a phenomenon first described by Warburg [34]. There also is compelling evidence that cancer cells are typically under greater oxidative stress compared to normal cells [35]. A difference between normal and cancer cells to oxidative stress that has been reported is the cytotoxicity caused by glucose deprivation. Studies by Spitz and coworkers [36] have clearly shown that this effect is mediated by mitochondrial ROS production.

The results described provide further evidence that a combination of sulindac and an oxidizing agent might have clinical therapeutic value in treating a variety of cancers. In a previous preliminary study we reported that the results of a limited proof of concept human clinical trial using sulindac (1–5%) and hydrogen peroxide (25%) gels applied daily for three weeks on actinic keratoses (AK) involving the upper extremities [27]. Upon completion, all ten treated AKs showed a reduction in size as shown by clinical photography with five exhibiting complete disappearance of the precancerous cells after skin biopsy. These preliminary results warrant more extensive clinical trials.

## Materials and Methods

### Materials

Sulindac, acetylsalicylic acid (aspirin), (S)-(+)-ibuprofen, and tert butyl-hydroperoxide (TBHP) were obtained from Sigma (St. Louis, MO). Sulindac sulfone was synthesized by Custom Synthesis Inc. (Boca Raton, FL). All tissue culture media including fetal bovine serum and other supplements were purchased from American Type Culture Collection (ATCC; Rockville, MD).

### Cell Culture

A colon cancer cell line (RKO), a lung cancer cell line (A549), and fibroblast cell lines derived from normal human colon tissue (CCD-18Co) and normal human lung (MRC-5) were obtained from ATCC (Rockville, MD). All cell lines were maintained in the recommended culture medium. The normal cell lines were not immortalized and early passage cells were used for the experiments reported here. Cell lines were determined to be free of mycoplasma using the VenorGeM® Mycoplasma Detection Kit (Sigma-Aldrich), which is a highly sensitive PCR-based assay.

### Cell Viability Assay

Unless otherwise indicated, the cells were pretreated with sulindac, sulindac sulfone or another NSAID for 48 hr prior to exposure to TBHP for 2 hr. Cell suspensions (∼100,000 cells) containing the indicated supplement were plated in 96 well microtiter plates using 100 µl of the indicated cell suspension. The plates were incubated for 48 hr at 37°C in a 5% CO_2_ incubator. The culture medium was then removed and the cells washed once with fresh culture medium with serum. After removal of the wash solution, fresh culture medium with serum that contained the indicated final concentration of TBHP or hydrogen peroxide was added to the cells, and the cells were incubated an additional 2 hours. Similar results were obtained when sulindac was included during the 2 hr treatment with the oxidizing agent.

Cell viability was determined by the CellTiter 96 Aqueous One Cell Proliferation Assay (Promega; Madison, WI) according to the manufacture's instructions. The assay utilizes a novel tetrazolium compound that metabolically active cells convert to a water-soluble formazan by the action of cellular dehydrogenases, which is measured by absorbance at 490 nm using a colorimetric microtiter plate reader (SpectraMax Plus^384^; Molecular Devices). Background absorbance was subtracted from each sample. It has been reported that some anticancer drugs can cause changes in absorbance in MTS-based assays for cell viability in the absence of cells [37]. Control experiments using sulindac alone, TBHP alone or combinations of sulindac and TBHP over the concentration range used in the reported experiments showed no effect of either compound alone or in combination on absorbance.

### Intracellular ROS Assay

Intracellular ROS levels were determined by using the Reactive Oxygen Species (ROS) Detection Reagents from Molecular Probes (Eugene, OR) as described elsewhere [38]. Elevated levels of ROS result in increased green fluorescence, which was visualized by fluorescence microscopy.

### JC-1 assay to measure mitochondrial membrane potential

Loss of mitochondrial membrane potential was determined using the JC-1 dye from Molecular Probes (Eugene, OR). Loss of mitochondrial membrane potential leads to increased green fluorescence in the cytosol and a corresponding decrease in mitochondrial red fluorescence. Thus, changes in mitochondrial membrane potential were determined by following the red to green staining shift using an FITC filter (Zeiss inverted microscope-Axiovert 40 CFL). Quantitation of the fluorescence signals used both standard densitometric methods and a Photoshop (Adobe Systems, San Jose, CA) based image analysis [39].

### TUNEL Assay to demonstrate apoptosis

Apoptotic cells were detected using the Deadend (Promega) colorimetric TUNEL assay that end labels fragmented DNA. Cells were fixed with 4% formaldehyde before being permeabilized with 0.2% triton-X-100. Cells were then washed with PBS prior to incubation with recombinant terminal deoxynucleotidyl transferase(TdT) and biotinylated nucleotides for 1 h at 37°C, which incorporates the biotinylated nucleotides at 3′ ends of fragmented DNA. Cells were washed with PBS and then incubated with horseradish peroxidase-streptavidin (HRP-Streptavidin) at room temperature for 30 min. HRP-streptavidin labeled cells were detected by hydrogen peroxide and diaminobenzidine (DAB). Apoptotic cells are visualized by dark brown nuclear staining.

### Statistical analysis

Results of cell viability experiments are expressed as the mean of four replicates of a representative experiment. The error bars indicate the standard error of the mean (SEM). Means were compared using standard t-tests and the P-values are indicated in the figure legends. P values<0.05 were considered statistically significant. Quantitation of the fluorescence signals used both standard densitometric methods and a Photoshop (Adobe Systems, San Jose, CA) based image analysis [39].

## Supporting Information

Figure S1 Cell Death due to Apoptosis. A TUNEL assay was used to detect apoptosis of lung cancer cells. (A) untreated cells; (B) cells treated with only 500 µM sulindac; (C) cells treated with only 180 µM TBHP; (D) cells treated with both 500 µM sulindac and 180 µM TBHP. Increased levels of apoptosis are indicated by enhanced formation of brown coloration. The experimental design is described in the legends of Figure 1 in the manuscript. Additional details of the TUNEL assay are provided in the Materials and Methods.
(0.22 MB TIF)
