# Supplementary figures and images for: Sulindac Enhances the Killing of Cancer Cells Exposed to Oxidative Stress
Source: PLoS One. 2009 Jun 5;4(6):e5804. doi: 10.1371/journal.pone.0005804 (PMC2686156; doi:10.1371/journal.pone.0005804)

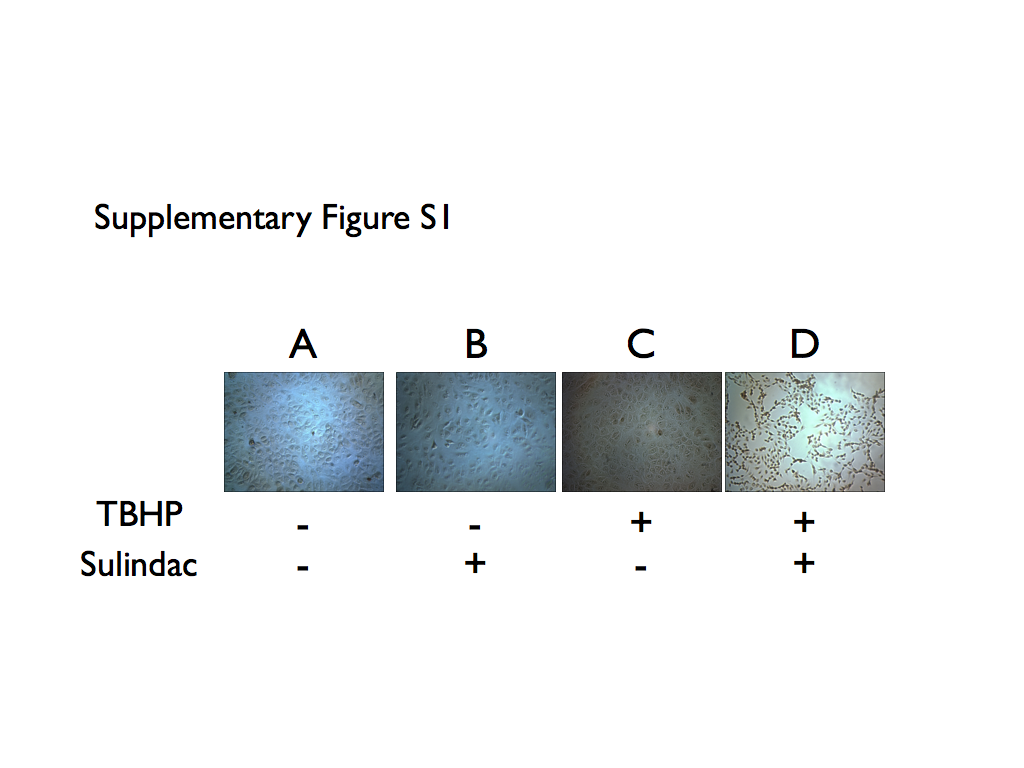

Supplement: Figure S1 — Cell Death due to Apoptosis. A TUNEL assay was used to detect apoptosis of lung cancer cells. (A) untreated cells; (B) cells treated with only 500 µM sulindac; (C) cells treated with only 180 µM TBHP; (D) cells treated with both 500 µM sulindac and 180 µM TBHP. Increased levels of apoptosis are indicated by enhanced formation of brown coloration. The experimental design is described in the legends of Figure 1 in the manuscript. Additional details of the TUNEL assay are provided in the Materials and Methods. (0.22 MB TIF) [file pone.0005804.s001.tif]
